# Age-specific incidence rates for cytogenetically-defined subtypes of acute myeloid leukaemia

**DOI:** 10.1038/sj.bjc.6600195

**Published:** 2002-04-08

**Authors:** A V Moorman, E Roman, R A Cartwright, G J Morgan

**Affiliations:** Leukaemia Research Fund Centre for Clinical Epidemiology, University of Leeds University, Leeds, UK; Department of Haematology, Leeds General Infirmary, Leeds, UK

**Keywords:** cytogenetics, chromosomal abnormalities, age, incidence rate, acute myeloid leukaemia, aetiology

## Abstract

It is generally considered that most cancers arise following the accumulation of several genetic events and that as a consequence its incidence increases with age. We report a cytogenetic subgroup of acute myeloid leukaemia whose incidence is independent of age. This observation indicates that acute myeloid leukaemia can develop via multiple pathways, and underlines the importance of cytogenetics in understanding this disease.

*British Journal of Cancer* (2002) **86**, 1061–1063. DOI: 10.1038/sj/bjc/6600195 www.bjcancer.com

© 2002 Cancer Research UK

## 

Cytogenetically-defined subtypes of acute myeloid leukaemia (AML) have distinct pathological and clinical features. In addition, many chromosomal abnormalities are important indicators of prognosis and are used to determine treatment strategies (Wheatley *et al*, 1999). Although, the link between cytogenetics and aetiology in AML remains uncertain some supporting evidence does exist. The main two cytogenetic subtypes seen in therapy-related leukaemia – 11q23 abnormalities and loss of 5q/7q – are closely associated with prior therapy with DNA topoisomerase II inhibitors and alkylating agents, respectively (Felix, 1998). Unfortunately, the results from the few studies investigating a link between smoking, alcohol, organic chemicals, pesticides and cytogenetic subgroups have been less convincing and are often contradictory (Crane *et al*, 1989; Sandler *et al*, 1993; Crane *et al*, 1996).

We, and others, have reported distinct age distribution profiles for AML patients with certain chromosomal abnormalities (IWCL4, 1984; Keating *et al*, 1987; Mauritzson *et al*, 1999; Moorman *et al*, 2001). Age is an important factor to be considered when investigating the aetiology of a disease as it can provide clues to its pathogenesis. The exponential increase in cancer incidence with age was fundamental to the development of the multi-stage theory of carcinogenesis because the probability of accumulating the full set of mutations required to effect malignant transformation increases with time (Armitage and Doll, 1954). In order to examine more closely the relationship between age and cytogenetics we have estimated the age-specific incidences rates of the principal cytogenetic subgroups seen in AML.

## PATIENTS AND METHODS

This study was based on cases with *de novo* AML recruited to a population-based case–control study of acute leukaemia (Kane *et al*, 1999). Briefly, the study ascertained adults (16–69 years old) diagnosed with acute leukaemia over a 5-year period in two regions of England. The Northern region consisted of the former Yorkshire Regional Health Authority and the counties of Lancashire and Cumbria. The Southern region was defined as the then regional health authorities of the South West and Wessex. Case ascertainment started in April 1991 and ended in March 1996 in the Northern region and in December 1996 in the Southern region. The current analysis has been restricted to patients with a pathologically confirmed diagnosis of *de novo* AML.

Diagnostic cytogenetic data were collected from regional laboratories and classified as previously described (Moorman *et al*, 2001). Cytogenetic analysis was considered successful if an abnormal clone or 10 normal metaphases were observed, otherwise it was classified as failed. Successful cases were classified hierarchically according to the type of the primary chromosomal abnormality into one of four karyotype groups: (1) translocations (including inversions and insertions); (2) deletions (including monosomies); (3) trisomies (including duplications); and (4) no abnormality detected (NAD). In addition, those cases with a chromosomal abnormality were also classified hierarchically according to the presence of the five most frequently observed specific aberrations: t(15;17)(q22;q12), t(8;21)(q22;q22), inv(16)(p13q22), del(5q)/−5/del(7q)/−7 and +8. Other abnormalities occurred too infrequently to be considered separately and were therefore grouped together under the ‘other’ heading.

The population resident within the study area was assumed to be stable during the study period and was estimated using 1991 UK census data, obtained from Manchester Information and Associated Services at the University of Manchester. Using age-specific population figures, the number of person-years in each region was calculated and summed to give the number of person-years in each age group. The study population was estimated at 7.8 million people, giving a total of 42.5 million person-years. Age-specific incidence rates were estimated (per million) by dividing the number of cases in each cytogenetic group by the number of person-years at risk. As AML is a rare disease the number of cases were not subtracted from the total.

## RESULTS

Of the 779 cases diagnosed with *de novo* AML 593 (76%) had a cytogenetic result, while 34 (4%) failed and 152 (20%) were not tested. Overall, the incidence of *de novo* AML increased with age – rising from <10 per million among those aged 16–29 years to >40 per million among those aged 60–69 years (Table 1

**Table 1:**
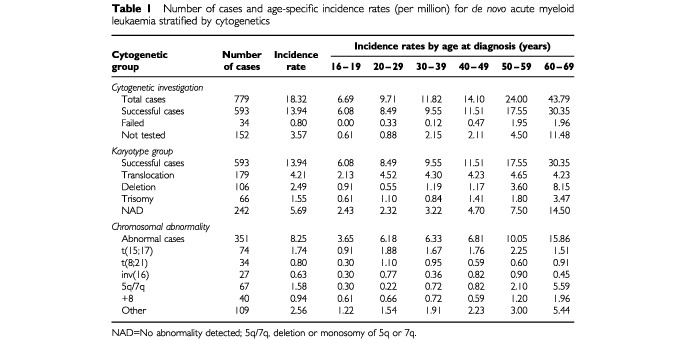
Number of cases and age-specific incidence rates (per million) for *de novo* acute myeloid leukaemia stratified by cytogenetics

). This pattern was observed for some but not all the cytogenetic subgroups investigated (Table 1, Figure 1

**Figure 1:**
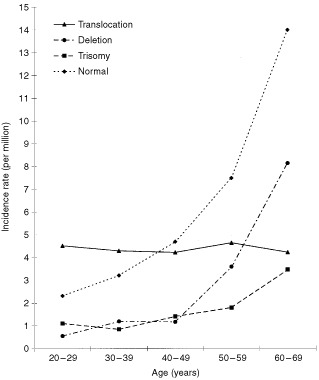
Age-specific incidence rates (per million) for *de novo* acute myeloid leukaemia by karyotype group.

). For example, the age-specific incidence rates for the deletion and normal karyotype groups as well as the 5q/7q group rose sharply with increasing age especially among the older two age groups. Although the incidence rates for the remaining three subgroups – trisomy, +8 and ‘other’ – also increased with age the rise was not as sharp and was confined to the oldest age group. The most notable exceptions to this pattern were the translocation karyotype group and the three specific translocations – t(15;17), t(8;21) and inv(16), whose incidence rates remained static across the different age groups.

## DISCUSSION

Our findings suggest that while the overall incidence of AML increases with age, the incidence of ‘translocation-positive’ AML does not increase with age. A recent study which showed that the incidence rate of acute promyelocytic leukaemia (which is synonymous with t(15;17)) is constant with age supports these findings (Vickers *et al*, 2000). It is generally assumed that cancer incidence increases with age because of the time required to accumulate the requisite number of mutations to effect malignant transformation. Armitage and Doll (1954) estimated that six or seven mutations would be required to produce the age distributions observed in their study. The age distribution of ‘translocation-positive’ AML clearly does not fit this model as its incidence is roughly constant between the ages of 16 and 69. Although we cannot calculate the actual number of mutations required to produce ‘translocation-positive’ AML from such an observation, it is reasonable to conclude that only one ‘rate-limiting’ step is required. Moreover, we can infer that there are at least two separate aetiological pathways which can give rise to AML. Therefore, studies investigating the causes AML should, where possible, assess the impact of cytogenetics on their findings.
